# Moderating and mediating mechanisms of the association between endogenous testosterone and aggression in youth: A study protocol

**DOI:** 10.1371/journal.pone.0319426

**Published:** 2025-02-25

**Authors:** Esther Calvete, Nerea Cortazar, Izaskun Orue

**Affiliations:** Department of Psychology, Faculty of Health Sciences, University of Deusto, Bilbao, Spain; Universidad de Concepción Facultad de Medicina: Universidad de Concepcion Facultad de Medicina, CHILE

## Abstract

**Background:**

The role of testosterone, particularly in combination with cortisol, in aggression remains debated. According to the general aggression model, hormones interact with other variables, such individuals’ knowledge structures, to predict aggression. This model may help explain inconsistent findings of previous research. Furthermore, the model posits that the influence of hormones on aggressive behavior is mediated by the individual’s internal state. Accordingly, the objectives of this study are to assess whether (1) testosterone and cortisol, alone and in interaction with aggression-related knowledge structures, predict aggression in a standardized laboratory paradigm and whether (2) this association is mediated by hostile attribution, anger, and arousal. By identifying key moderators and mediators, this study seeks to make a very novel contribution to the understanding of the role of testosterone in aggressive behavior. These objectives will also be assessed separately for males and females.

**Methods:**

The Point Subtraction Aggression Paradigm will be used to assess aggression. The sample will include 110 youths aged 17–19 (50% male). Participants will provide saliva samples before and after completing the paradigm, along with measures of three aggression-related knowledge structures (justification of violence, hostility, and narcissism) collected before the paradigm. Measures of two mediators (hostile attribution, anger) will be obtained after completing the paradigm. Arousal levels (heart rate and skin conductance) will be recorded before, after, and while completing the paradigm. The hypotheses will be tested using path analysis models, examining both the testosterone/cortisol ratio and the interaction between testosterone and cortisol as well as both baseline hormone levels and changes in hormone levels.

**Discussion:**

Although it may be limited by the use of saliva for measuring hormones, this study will enhance the current understanding of the role of testosterone and cortisol in aggressive behavior among youths by investigating novel hypotheses related to psychological moderating and mediating factors.

## Introduction

For many years, testosterone has been considered an important factor in explaining social status-related behaviors, including aggression [1]. Levels of testosterone, a hormone produced by the hypothalamic–pituitary–gonadal (HPG) axis, increase significantly during adolescence, particularly in males [2]. However, its role in human aggression has been widely debated, as several reviews and meta-analyses have failed to reach a definitive conclusion regarding this relationship [2–4]. Some research has suggested that the effects of testosterone may be moderated by cortisol levels, as proposed by the dual hormone hypothesis (DHH) [5]. However, studies investigating the joint influence of testosterone and cortisol have also yielded inconclusive results [6].

To address the above research gap, this study aims to extend previous research by introducing innovative hypotheses and methodological improvements to provide a more comprehensive understanding of the role of testosterone in aggressive behavior among youths. Specifically, the study posits that the relationship between testosterone (and hence cortisol) and aggression may be moderated by psychological variables derived from the general aggression model (GAM) [7,8], a prominent framework for aggressive behavior in humans. According to the GAM, hormones do not operate in isolation; rather, psychological factors known as knowledge structures (KSs) may moderate the effects of both testosterone and cortisol.

Additionally, the study will explore the mediating mechanisms underlying the association between testosterone (and cortisol) and aggressive behavior. Following the GAM, it is proposed that the interaction between hormones and knowledge structures predicts an individual’s internal state, which comprises three key components: cognition, affect, and arousal. Using a rigorous laboratory methodology, this study will examine the extent to which these elements contribute to explaining the role of testosterone and cortisol, in combination with other psychological characteristics, in the resulting aggressive behavior. Thus, by combining psychological and hormonal variables, this study offers a very novel approach to understanding the role of testosterone in human aggression. In doing so, the study seeks to make a significant contribution to the understanding of testosterone’s role in aggressive behavior by identifying critical moderators and mediators within this relationship.

### Role of testosterone and cortisol in the association between testosterone and aggression

As mentioned above, despite years of research on the role of testosterone in aggression, studies and meta-analyses have failed to clearly establish an association [2,4]. Consequently, the role of this hormone in human aggression remains controversial. For example, in a meta-analytic review [4], the authors concluded that the associations between endogenous testosterone (both basal levels and situational changes) and aggression are relatively weak. They partly attributed these results to methodological issues. Thus, for example, while most studies have focused on baseline levels of testosterone, others have attempted to capture the dynamic nature of testosterone, focusing on fluctuations in testosterone in response to some type of environmental provocation. Geniole et al. [4] found that aggression was very weakly associated with baseline testosterone and that it tended to be somewhat more strongly associated with changes in testosterone levels. In addition, both indicators of testosterone were only significantly associated with aggression in male samples. Moreover, the authors found that most studies have assessed aggressive behavior using self-reports, which are more susceptible to response bias than behavioral measures [9]. Thus, the use of different methodologies may have contributed to the lack of conclusive results.

More importantly, it has been proposed that cortisol may moderate the association between testosterone and aggressive behavior. When a person is under stress, the hypothalamic–pituitary–adrenal (HPA) axis secretes cortisol [10]. Although the specific role of cortisol in aggressive behavior remains unclear [11–14], it has received considerable interest due to its potential moderating effect on testosterone in the context of aggressive behavior. The DHH [5] posits that testosterone is associated with aggressive behavior only when cortisol levels are low. Conversely, when cortisol levels are high, there is no such relationship between testosterone and aggressive behavior [15]. Although this theory is intriguing, empirical support is limited. For instance, the DHH received only moderate support in a meta-analytic evaluation [6], with a very small effect size for the interaction between baseline levels of testosterone and cortisol on aggressive and status-related behaviors. Moreover, the authors noted that the effects were even smaller in women [6]. It should be noted that their meta-analysis reported similar methodological limitations in previous research to those found in studies solely focused on testosterone.

Moreover, some studies have found interaction patterns between testosterone and cortisol which, while significant, differ from those proposed by the DHH. For instance, among a sample of women who were insulted and then given the chance to respond aggressively to the aggressor, baseline testosterone was found to positively predict reactive aggression only among participants with high baseline cortisol levels [16]. Another study involving a large sample of undergraduate students found that, in women but not in men, reactive aggression was higher when both baseline cortisol and testosterone levels were low and when baseline cortisol levels were low and change in testosterone levels was high [17]. Similar outcomes were found in a sample of adolescents, with those exhibiting either elevated baseline testosterone and cortisol levels or low baseline testosterone and cortisol levels demonstrating increased aggression in response to peer victimization [18]. In a longitudinal study involving children and adolescents conducted over three time points with a six-month interval, it was found that combinations of low diurnal testosterone levels and low cortisol reactivity as well as high diurnal testosterone levels and high cortisol reactivity were associated with increased antisocial behavior and oppositional defiant disorder symptoms in boys [19]. However, the results for girls were not statistically significant. Consequently, the general findings for the DHH are inconclusive. Furthermore, current evidence suggests that the interplay between testosterone and cortisol may be different for men and women.

Moreover, some previous studies on the DHH have chosen to examine the testosterone/cortisol ratio (T/C ratio) rather than the interaction between testosterone and cortisol [10]. Specifically, it has been suggested that a higher T/C ratio (i.e., high testosterone relative to cortisol) is associated with aggressive behavior. These two ways of evaluating DHH appear to lead to different results. For example, in a non-clinical sample of adolescents, a positive T/C ratio was found to be associated with aggressive behavior, whereas the interaction between testosterone and cortisol was not significantly related to aggressive behavior [20]. However, the use of the T/C ratio has been criticized because it does not enable capturing the effects of all possible testosterone and cortisol combinations [18,21].

### Moderating role of knowledge structures in the action of testosterone and cortisol

The GAM [7,8] proposes that hormones do not act in isolation but are influenced by situational (i.e., provocation) and individual (e.g., KSs) variables. KSs are internal mental representations of past experiences and behaviors and determine the way in which people perceive, categorize, and interpret situations [22]. Previous studies have identified an association between certain KSs, such as justification of violence, narcissism, and hostility/mistrust, and aggressive behavior [23]. Justification of violence refers to normative beliefs about the social appropriateness of aggression [24]. These beliefs encompass the idea that the use of aggression is justified (e.g., because the other deserves it) and that it leads to positive outcomes for the individual (e.g., because it serves to gain respect from others). Numerous studies have linked this type of KS to both reactive and proactive aggressive behavior [23,25] as well as to bullying [26,27]. The hostility or mistrust KS is defined as the expectation that others will inflict harm, abuse, or take advantage of one—and that such harm is intentional [28]. Multiple studies have shown that this KS is associated with aggressive behavior in adolescents [23,29]. Finally, narcissism is defined as the belief that one is superior to others and deserving of special rights and privileges. Several longitudinal [30] and experimental studies [31] have demonstrated a relationship between narcissism and aggression. Moreover, this relationship is observed to be stronger during provocation, although it is significant for a range of different forms of aggressive behavior, including proactive, reactive, bullying, and physical and verbal aggression, as evidenced by recent meta-analyses [32,33].

Although no previous studies have explicitly analyzed the interaction between the three KSs described above and testosterone and cortisol, evidence from research on other individual traits and these hormones suggest that such interactions may be plausible. For example, Geniole et al. [34] reported that testosterone was related to aggressive behavior in men with specific personality profiles characterized by high dominance, which is related to narcissism, independent self-construal, and low self-control. With regard to cortisol, a recent systematic review concluded that some psychological factors can moderate the relationship between cortisol and aggressive behavior [35]. The authors recommended a comprehensive biopsychosocial approach, which combines studies of hormones and other psychological traits (e.g., empathy, prosociality) to gain a better understanding of youth aggression [35].

Notably, in one of the few studies assessing the three-way interaction of testosterone x cortisol x a psychological trait, Grotzinger et al. [36] found two three-way interactions between testosterone, cortisol, and both peer deviance and peer prosociality, constructs that are similar to the justification of violence and narcissism KSs. Their findings indicated that, at high levels of peer deviance, testosterone predicted increased rule breaking behavior at elevated levels of hair cortisol. Conversely, under high peer prosociality, testosterone predicted increased rule breaking behavior at lower levels of cortisol.

### Mediators: Possible explanatory mechanisms for the action of testosterone and cortisol

The GAM [7,8] posits that the influence of biological variables and KSs in aggression could be mediated by the individual’s internal state, which includes experiences of affect (e.g., anger), cognition (e.g., hostile thoughts), and arousal (e.g., heart rate and skin conductance). However, research on these mediational mechanisms is scarce. Regarding the association between testosterone, cortisol, and anger, the few available studies have reported mixed results. For instance, in one study, exogenous testosterone administration was associated with increased anger in response to a provocative situation, and this increased anger mediated the relationship between testosterone and an implicit measure of aggression [37]. Further, the T/C ratio has been shown to be cross-sectionally related to anger expression in individuals who perpetrate intimate partner violence [38]. In a study using the cyberball task, change in testosterone but not in cortisol levels was found to be associated with anger in students [39]. Finally, in a sample of children, high levels of testosterone and cortisol were cross-sectionally associated with both low and high levels of anger [40].

The second potential mediating mechanism proposed by the GAM is hostile attribution. Hostile attribution is an important element of the social information processing model [41], which was integrated into the GAM to explain aggressive behavior. According to these models, individuals who interpret the intentions of others as hostile in ambiguous situations will be more likely to respond aggressively. Several studies have established the relationship between hostile intent and aggressive behavior [42]. Although little research has specifically investigated the association between testosterone, cortisol, and hostile attribution, the results of a study involving a sample of juvenile offenders supported the DHH and the GAM, as hostile attention bias mediated the association between baseline testosterone levels and aggression, while baseline cortisol levels moderated that association [43]. Specifically, at a high cortisol level, testosterone positively predicted attention bias away from hostile stimuli, thereby reducing the aggression level.

Finally, arousal, which may involve increased sympathetic nervous system (SNS) activation and manifest in indicators like increased heart rate and electrodermal reactivity, is the third mediating mechanism proposed by the GAM. It is important to note that both very high and very low levels of arousal may be associated with different types of aggressive behavior (i.e., reactive vs. proactive aggression) [35] and that there is high variability in results depending on factors like sample type [44]. For example, in a study that examined the relationship between cardiovascular reactivity and reactive and proactive aggression among women, less reactivity was associated with proactive relational aggression, while exaggerated reactivity was associated with reactive relational aggression [45]. In another study on university students, baseline skin conductance was related to proactive aggression in women, while skin conductance in response to stress was positively related to reactive aggression and negatively related to proactive aggression in men [46]. However, there is scant research on the link between hormones and SNS reactivity. In one study, higher SNS activation, as indicated by high skin conductance reactivity, was found to be associated with baseline testosterone levels in individuals who perpetrated intimate partner violence [47].

### The present pre-registered study

This study is part of a larger project investigating factors that moderate and mediate the influence of testosterone and cortisol on aggression in adolescents and young adults (TESCOR, Ref. PID2022-140773NB-I00). The project incorporates school-based studies using hormonal measurements and self-reports, complemented by the present laboratory study, which focuses on behavioral measures of aggression and psychophysiological indicators of arousal.

The main objective of this study is to address important research gaps in understanding the psychological variables that moderate and mediate the effects of testosterone and cortisol on adolescent aggressive behavior when they are provoked. This objective is structured around two research questions.

Research question 1: Do aggression-relevant KSs (mistrust/hostility, narcissism, and justification of violence) moderate the association between testosterone and cortisol and aggressive reactions when youths are exposed to an experimentally manipulated provocation? No previous studies have examined the interaction between these KSs and hormones in aggressive behavior, although a few have examined the role of other related traits [34,36]. As different methodologies have been employed in previous studies, several will be employed in this study. Both baseline levels and changes in testosterone and cortisol levels will be measured, as previous research has reported different results for both indicators [4]. Additionally, hypotheses will be tested using models that consider the interaction between testosterone and cortisol and the T/C ratio. Instead of employing measures of self-reported aggression, this study will employ behavioral measures, which are less biased [9]. It is hypothesized that the association between both baseline levels and changes in levels of testosterone and cortisol and aggression will be stronger at higher KSs levels. However, given inconsistent prior findings [18,19], specific hypotheses about which combinations of testosterone and cortisol will be most relevant to aggression are not made.

Research question 2: Do anger, hostile attribution, and arousal (heart rate and skin conductance) mediate the relationship between testosterone, cortisol (alone and in interaction with KSs), and aggressive behavior in response to an experimentally manipulated provocation? This important topic remains underexplored. One study found that hostile attention bias mediated the association between testosterone and aggression and that baseline cortisol moderated that association [43]. Other research has associated testosterone with increased skin conductance [47], and it has been reported that testosterone and cortisol are cross-sectionally associated with anger [38] and that change in testosterone levels during the cyberball task is associated with anger [40]. In this study, consistent with the GAM, a mediated moderation model is hypothesized in which the joint action of hormones and KSs on aggressive behavior is mediated by three components of the individual’s internal state: hostile attribution, anger, and arousal.

Gender differences are a secondary focus, as prior research suggests the effects of testosterone and cortisol are more pronounced in men than women. However, due to the high costs of obtaining sufficiently large subsamples of men and women for biological and psychophysiological measures, gender-based analyses will be exploratory, examining potential model invariance.

## Materials and methods

### Participants

This study focuses on aggressive behavior in late adolescents. To facilitate laboratory attendance, the sample will consist of first- and second-year college and vocational training students (17–19 years old; late adolescence). A larger pool of late adolescents (N ≈  350) will be invited to participate, from which the final sample (N ≈  110) will be selected based on KSs levels related to aggression (low, medium, and high) to ensure adequate distribution. In addition, the sample will be gender balanced (approximately 50% male). Details on sample estimation are provided in the data analysis section.

#### Inclusion and exclusion criteria.

Inclusion criteria will be healthy adolescents between 17 and 19 years of age interested in participating in a study on the role of hormones in the context of a videogame in which they can win or lose points, who have sufficient knowledge of Spanish or Basque to understand the questionnaires and instructions. Exclusion criteria will include having any cardiovascular disease, taking any medication that affects the central nervous system, or taking testosterone or cortisol hormone supplements.

### Paradigm

The Point Subtraction Aggression Paradigm (PSAP) [48] will be used in this study. The PSAP is an online computer game where participants compete against a fictitious opponent. Participants are informed that the goal is to earn as many points as possible and that the points earned can be exchanged for money at the end of the task (monetary incentive). To earn points, participants must press a designated key on the keyboard a certain number of times consecutively (e.g., one point for every 100 consecutive keystrokes). Participants are also informed that their point counter may flash red and decrease in response to the other player stealing a point from them (i.e., provocation). They can respond to this provocation in three ways: (1) continue earning points by pressing the same key, (2) press a second key to protect their points for a variable amount of time, or (3) press a third key to steal points from the opponent. However, stealing does not increase the participant’s point total; it only reduces the opponent’s points.

The paradigm can be adapted to different durations. In this study we will use the tasks developed in E-PRIME® software [49]. Participants will complete three 10-minute tasks under varying levels of provocation. In the first trial (Run 1), the participants will be provoked 10 times and lose a total of 10 points. In Run 2, they will be provoked 20 times and lose a total of 20 points. In Run 3, they will be provoked 10 times and lose a total of 20 points. Following [49], we will use an inter-press interval of 50 ms.

### Design

We will use a prospective design with multiple causal steps [50]. In this design, a group of variables (hormones and knowledge structures) will be examined as predictors of changes of other variables (internal state variables), and these in turn will be examined as predictors of aggressive behavior. Therefore, variables act as outcomes and predictors at different points in the hypothesized sequence.

### Measures

#### Outcome variable.

Aggressive behavior will be measured as the number of points stolen during the PSAP. The PSAP has demonstrated validity through correlation with other measures of aggression [51] and high test–retest reliability [52]. Different scores may be obtained (e.g., percentage of steal presses or steal presses, controlling for earn and protective presses). Although the focus will be on reactive aggressive behavior (i.e., during the provocation intervals), additional analyses will be conducted on proactive aggressive behavior. For this purpose, the thefts made by the participant during the period prior to the first provocation in each trial (45 seconds) will be used as an indicator of proactive aggression [51].

#### Predictors.

Testosterone and cortisol will be measured at baseline and after completing the paradigm to obtain indicators of baseline and change. In this study, saliva will be used to obtain hormonal indicators because, although only the free fraction of steroid hormones can be measured in saliva, their concentrations in saliva are usually proportional to hormone levels in blood and saliva sampling is much easier and relatively less stressful for research participants than blood sample collection. Thus, salivary hormone assessment has become the method of choice among psychoneuroendocrinologists working with human populations [53]. On the day of salivary sample collection, the participants will be instructed to avoid brushing their teeth, consuming a meal, or drinking at least 60 minutes prior to sample donation. They will also be asked to avoid intense exercise eight hours prior to data collection. The research team will confirm that each participant has followed the guidelines. If the guidelines have not been followed, the participant will be invited to attend on a different day. The samples will be stored in a freezer (−80ºC) until assayed. Saliva samples will be assayed for testosterone (pg/ml) and cortisol (nmol/L) levels in duplicate determinations to further enhance the reliability of the data. We will utilize an enzyme-linked immunosorbent assay (ELISA), a validated and widely used method in hormone research due to its robustness, high sensitivity, and specificity, for quantifying testosterone and cortisol levels. ELISA is suitable for reliably measuring hormone concentrations in body fluids, including saliva. We will use commercially available assay kits, which are rigorously validated to ensure accuracy and reproducibility.

Three KSs will be assessed: justification of violence, mistrust/hostility, and narcissism. The justification of violence schema will be measured with the nine-item Justification of Violence subscale of the Irrational Beliefs Scale for Adolescents [54]. The other two KSs will be assessed using the corresponding subscales (five items each) of the Young Schema Questionnaire-3 [55], adapted to Spanish [56]. The response scale ranges from 1 (*completely untrue of me*) to 6 (*describes me perfectly*). Alpha coefficients for these measures in previous research have been adequate (e.g., .78–.82) [23]. Previous research has indicated that the correlations between these KSs tend to be moderate to high [23,27]. Therefore, a composite score will be generated through factor analysis.

Finally, several sociodemographic variables will be measured, including age, gender, and socioeconomic status.

#### Mediators.

Anger will be assessed with the Scale for Mood Assessment [57], which measures transitory moods. The four items that form the anger subscale will be used. The items are answered on an 11-point scale, ranging from 0 (*nothing*) to 10 (*a lot*), indicating how respondents feel at the moment. Alpha coefficients for the anger subscale in previous research ranged from .93 to .95 [58].

Hostile attribution will be assessed using an adaptation of the procedure employed by Hiemstra et al. [59], consisting of two items—ranging from 0 (*completely not agree*) to 4 (*completely agree*)—which assess the negative intentions of the person who allegedly steals points from them: “He/she wanted to annoy me by stealing my points” and “He/she did it to show me that I’m worse than him/her.”

Arousal indicators, including heart rate (HR) and skin conductance level (SCL), will also be assessed. An electrocardiogram (ECG) will be used to measure HR, while electrodermal activity will be used to measure SCL. For this purpose, the BioPac M150 system will be used at a sampling rate of 1000 Hz and processed in AcqKnowledge^®^ 5.0. We will use lead II—with the negative electrode on the right clavicle and the positive one on the last left rib—with disposable foam electrodes (after rubbing the skin with cotton and skin preparation gel) for the ECG. Following the standards for electrodermal measurements [60], SCL will be recorded with electrodermal electrodes using isotonic electrode paste. Electrodes will be placed on distant phalanges of the ring and index fingers of the participant’s non-dominant hand, and SCL will measured in microsiemens. Prior to preprocessing, all data will be visually screened for artifacts (AcqKnowledge^®^ 5.0). HR and SCL will be estimated using the default processing settings in AcqKnowledge^®^ 5.0. HR and SCL will be obtained in the following sequence: baseline (5 minutes), three runs of the PSAP (10 minutes each), and recovery (5 minutes).

### Procedure

**Fig 1:**
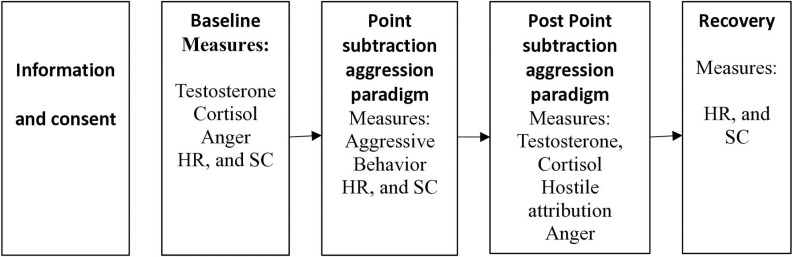
Design and steps of the study.

Selected participants will be invited individually to the laboratory to complete the study. The procedure will consist of the following steps: (1) Information and consent: The participant will be informed about the details of the experiment, the inclusion and exclusion requirements will be checked, and if they are met, the participant will sign an informed consent form. (2) Baseline measures: A saliva sample will be collected to measure testosterone and cortisol levels, after confirming that the saliva collection guidelines have been met. The participant will complete the anger measure, and then arousal indicators (HR and SCL) will be recorded for five minutes. (3) PSAP: The participant will be asked to perform three 10-minute online computer game behaviors. The number of aggressive behaviors (stealing) during each run will be recorded. Additionally, arousal measures will be recorded continuously during each 10-minute run. (4) Post PSAP measurements: A second saliva sample will be collected, and the participant will complete the anger and hostile attribution measures. (5) Recovery: At the end of the session, arousal measures will be recorded again for five minutes. Next, the participant will be thanked for participating, invited to ask any questions, and provided with a voucher. Fig 1 depicts the phases of the study.

### Data analysis

Since the whole process will be carried out in a single session, significant missing data are not anticipated, aside from potential issues with psychophysiological recording. Nevertheless, Little’s test of Missing Completely at Random (MCAR) will be estimated, and if statistically significant, multiple imputation (*N* =  100 samples) will be used to address missingness. In the event the MCAR results are not statistically significant, the full information maximum likelihood technique will be used. Outliers in the study variables will be winsorized at 3 standard deviations above the mean. Variables that are not normally distributed will be logarithmically transformed. In addition, all variables will be transformed into *z*-scores before creating interaction terms.

**Fig 2:**
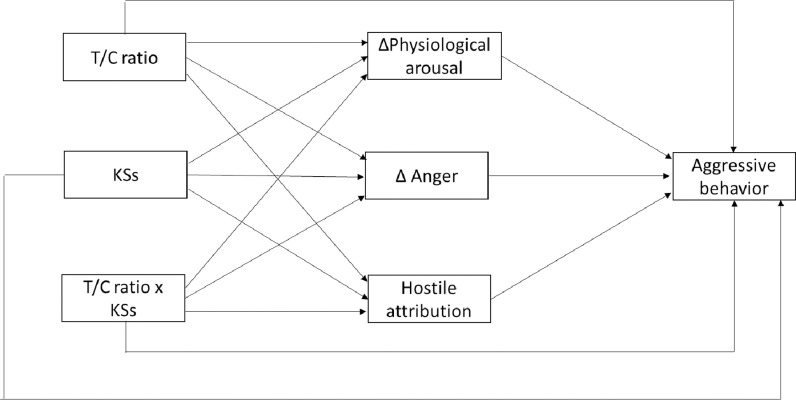
Model 1: Baseline T/C ratio as a predictor of aggressive behavior.

**Fig 3:**
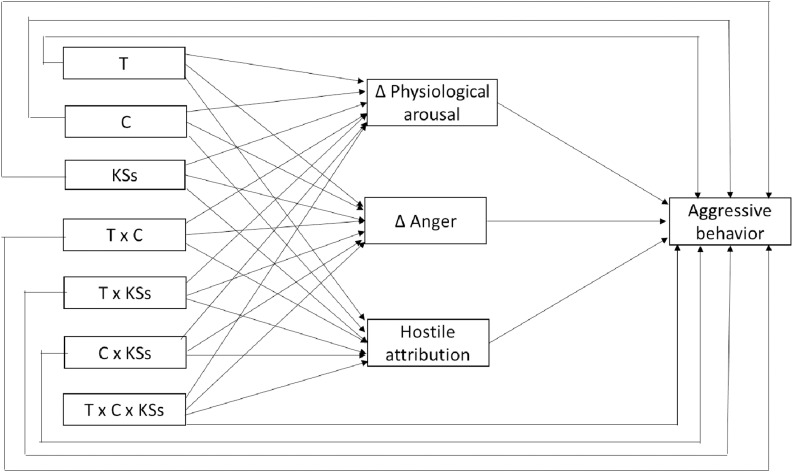
Model 2: Baseline T and C levels and their interaction as predictors of aggressive behavior.

We will estimate four path analysis models with MPLUS 8.10 to test the hypotheses. Model 1 will focus on the baseline T/C ratio (Fig 2). In this model, the T/C ratio, KSs, and the interaction between the T/C ratio and KSs will be included as predictors of aggressive behavior both directly and indirectly through three mediators (arousal, anger, and hostile attribution). In order to reduce the number of parameters in the model, arousal will reflect the change in psychophysiological indicators (HR and SCL) between baseline and during the task (posttest), while anger will reflect the increase in this variable from baseline to posttest. Model 2 will be similar to model 1, but the change in the T/C ratio will be used. Model 3 will use testosterone and cortisol levels at baseline as predictors as well as KSs and their interactions as predictors (see Fig 3). Finally, model 4 will be similar to model 3 except that it will use the change in testosterone and cortisol levels from baseline to posttest as a predictor. Table 1 presents the variables used in each model. As shown in Figs 2 and 3, the models will include both direct and indirect effects between predictors and aggressive behavior. In addition, residual covariances between mediators will be included in all models. The models described will focus on reactive aggressive behavior. However, they will be replicated for proactive aggressive behavior (i.e., the thefts made by the participant during the period prior to the first provocation in each trial).

**Table 1:** Summary of the variables included in the study models.

	Predictors	Mediators	Outcome
Model 1 Baseline T/C ratio	T/C ratio KSs T/C x KSs	Increase in physiological arousal Increase in anger Hostile attribution during the task	Aggressive behavior
Model 2 Change in T/C ratio	Change in T/C ratio KSs Change in T/C x KSs	Increase in physiological arousal Increase in anger Hostile attribution during the task	Aggressive behavior
Model 3 Baseline T and C interaction	T C KSs T x C T x KSs C x KSs T x C x KSs	Increase in physiological arousal Increase in anger Hostile attribution during the task	Aggressive behavior
Model 4 Change in T and C interaction	∆T ∆C KSs ∆T x ∆ C ∆T x KSs ∆C x KSs ∆T x ∆ C x KSs	Increase in physiological arousal Increase in anger Hostile attribution during the task	Aggressive behavior

Note. T = Testosterone, C = Cortisol, KSs = Knowledge Structures

Model fit will be assessed using the comparative fit index (CFI), Tucker–Lewis index (TLI), root mean square error of approximation (RMSEA), and standardized root mean square residual (SRMR). RMSEA values ranging from .02 to .05 reflect a good fit, while those between .05 and .08 represent an acceptable fit [61]. For CFI and TLI, values between .95 and .99 suggest excellent fit, whereas values from .90 to .95 denote acceptable fit. Finally, SRMR values below .08 are considered an indicator of adequate fit to the data [62].

Finally, we will examine the invariance of the models described above for men and women. For this purpose, we will estimate configural models in which all path coefficients are freely estimated for each group and invariant models in which these coefficients are set as equal. Changes in χ^2^ and in CFI between models will be used as indicators of whether the invariance of the models is acceptable, considering criteria proposed by several authors [63,64].

Fully anonymized data will be uploaded to Open Science Framework (OSF) to be permanently accessible for consulting purposes and to obtain data for meta-analysis (Doi: 10.17605/OSF.IO/WY94K). We aim to publish all results from this study in peer-reviewed international journals and to disseminate the findings at international scientific conferences.

#### Sample determination.

Sample size was estimated via Monte Carlo simulation [65]. This approach involves defining a model in which the values of the parameters are determined from theory or previous research. The model is specified as true for the population, and then a large number of samples of constant size are drawn from this population. We calculated the model parameters in each of the samples and estimated a probability statement about the proportion of all replications that included a path coefficient that reached significance at the .05 level. This proportion represents the empirical power estimate. We followed the recommendations of Thoemmes et al. [66], who adapted the Monte Carlo approach to mediational models, to calculate the power for the total, direct, and indirect effects.

In setting initial values for the parameters in the population, values representing small effect sizes were chosen for associations between aggressive behavior and hormonal and psychophysiological measures, while values representing medium effect sizes were selected for associations between self-reports (e.g., anger and KSs) and between self-reports and aggressive behavior. Models 1 and 2, which are the simplest, include 54 parameters. Since a minimum number of 55 participants in each subgroup is needed for model comparison in samples of men and women, Monte Carlo was calculated for a total sample of 110 participants. The power for the total effects of T/C ratio, KSs, and T/C ratio x KSs range between 97 and 100% in the total sample and between 78 and 98% in the subsamples of men and women.

In models 3 and 4, with a sample of 110 participants, the statistical power for the total effect would range between 93% (testosterone x cortisol) and 98% (for KS). However, given that the number of parameters in these models is considerably higher (i.e., 77) than in models 1 and 2, the minimum sample size for estimating the models is 78 participants, so a total sample of 156 participants would be needed to perform the sex invariance analyses. Given the high cost of obtaining a sample of this size, the choice has been made to estimate models 3 and 4 with one mediator at a time for the sex invariance tests, reducing the number of parameters to 54, and the estimation of the power statistic will be similar to that of models 1 and 2. Post hoc power analysis will confirm the findings.

### Ethical considerations

The study has been approved by the research ethics committee of the University of Deusto (Ref. ETK-28/22-23). Participants must sign an active informed consent form. Confidentiality will be maintained, and data will be protected. Based on the evaluations of previous research ethics boards [51], the participants will be provided with the same monetary sum at the end of the game (20 euros), a sum that is greater than the maximal exchange possible based on the specific version of the PSAP used. The researchers will be psychology graduates with experience in studies on aggressive behavior and hormones. If participants show signs of distress during the task, they will be reassured and given the opportunity to withdraw from the study. However, this paradigm has been used in numerous studies, and no such incidents have been reported among participants. Some participants may experience anger, but enjoyment is also commonly observed during the task [51]. Since the study involves a certain degree of deception, all participants will receive a debriefing at the end of the study.

## Discussion

After decades of research on the association between testosterone and aggression, meta-analyses and reviews indicate that the results remain inconclusive [2,4]. Previous research has also suggested that the role of testosterone may be moderated by levels of cortisol, giving rise to the DHH model [5]. However, as has been the case with studies focused exclusively on the role of testosterone, studies assessing the joint action of testosterone and cortisol have proven to be inconclusive [6].

The current study extends previous research by proposing innovative hypotheses to provide a more compressive understanding of the role of testosterone, both independently and in combination with cortisol, in aggressive behavior. Specifically, the project uses the GAM as a framework [7,8], one of the most important models of human aggressive behavior. It hypothesizes that KSs related to aggression may moderate the relationship between testosterone, cortisol, and aggression. No previous studies have examined the role of these KSs, despite their potential to shape the interpretation of social confrontations, such as provocation or justified aggression. By interacting with hormonal levels and changes in these hormones, these KSs may play a critical role in aggressive outcomes.

The study also aims to identify mediating mechanisms that help explain the link between testosterone (and cortisol) and aggressive behavior. Following the GAM, it is proposed that the interaction between KSs and levels of testosterone and cortisol predict an individual’s internal state, comprising three key components: cognition, affect, and arousal [8]. Using a rigorous laboratory study, this study will examine the extent to which these elements contribute to explaining the role of testosterone, in combination with cortisol levels and KSs, in the resulting aggressive behavior. In doing so, this study will address an important but neglected area of knowledge. The few previous studies that have examined the possible mediational role of the internal state components proposed by the GAM have focused on anger [38,40]. For example, one study assessed the cross-sectional association between anger and testosterone and cortisol levels [38], while another assessed the association between anger and change in testosterone [39]. Our study will expand on these studies by including measures of both baseline levels and changes in hormone levels in a prospective laboratory study. Previous research including other internal state dimensions (i.e., hostile attributions and arousal) is even scarcer [43,47]. Consequently, this study has significant potential to contribute to understanding the mechanisms underlying the effects of testosterone and cortisol on aggressive behavior.

### Strengths of the study

From a methodological standpoint, this study addresses limitations of prior research. Unlike previous studies that predominantly relied on baseline testosterone and cortisol measurements [16,18,20], this study will integrate baseline and reactive testosterone and cortisol levels following provocation. While a few exceptions exist [17,19], such approaches are uncommon in the literature.

Another strength is that the study will use the PSAP, a validated measure of reactive aggressive behavior [51]. Thus, it will improve upon prior studies that relied solely on self-reported measures of aggression [17–20].

### Risks and limitations

This study is not without risk. A key challenge lies in achieving the necessary sample size to ensure adequate statistical power. Many previous studies have suffered from insufficient statistical power (for exceptions, see [17,18,20], especially when comparing subsamples of men and women [4]. Moreover, this is a costly study due to its individualized protocol, which includes biological sampling, psychophysiological measurements, and financial compensation for participants. Thus, it will be important to estimate the minimum sample size needed. The Monte Carlo approach was used for this purpose. To reduce the number of model parameters and thereby reduce the required sample size, change scores will be used as mediating variables instead of repeated measures. While this solution is useful for parameter reduction, it is suboptimal from an analytical perspective, requiring post hoc analyses using alternative data analysis strategies.

Another challenge relates to recruiting a balanced sample of male and female participants. Since prior research indicates that the role of testosterone is more pronounced in males than in females [4,6,17], achieving gender parity in the sample is critical. However, recruiting male participants in this age group has proven difficult in past studies, presenting a logistical hurdle.

Finally, this study is not without limitations. The main limitations of the study relate to the use of saliva to obtain indicators of testosterone and cortisol since only the free fraction of steroid hormones can be measured in saliva. However, the use of blood samples can be extremely stressful for many participants and saliva sampling offers a convenient alternative while its results are consistent with those obtained with other methods [53]. Another limitation of the present study concerns other factors that may influence the role of testosterone. Many of testosterone’s behavioral effects are partially mediated by androgen receptors, which may be expressed in brain regions such as the amygdala [67]. The function of androgen receptors depends, for instance, on trinucleotide repeat (CAG) polymorphism, where a lower number of CAG repeats increases testosterone’s efficacy in exerting its effects through the androgen receptor. For example, a lower number of CAG repeats has been positively linked to aggressive behavior [68]. Therefore, future research could include DNA analysis to genotype the androgen receptor CAG repeat polymorphism.

## Conclusions

This GAM proposes that hormones interact with psychological variables such as knowledge structures related to aggression and that the role of hormones and knowledge structures in aggressive behavior is mediated by the internal state of the individual. Despite the influence that GAM has had in the last decades in the understanding of human aggression, surprisingly its application to shed light on the role of testosterone in aggressive behavior had not been studied until now. In this innovative study, we apply the GAM to explain the role of testosterone on aggressive behavior. Furthermore, it will enable us to ascertain the manner in which both testosterone and cortisol interact with individual variables, such as KSs. In this way, the study can help clarify some of the mixed results obtained in previous research regarding the role of these hormones in aggressive behavior in humans. Finally, the study will contribute to identifying the mediating role of proximal factors in provocation scenarios, including hostile attribution, anger, and arousal. This knowledge will inform the development of more effective interventions to prevent and address aggressive behavior among youths.
